# Rapid identification of mutations caused by fast neutron bombardment in *Medicago truncatula*

**DOI:** 10.1186/s13007-021-00765-y

**Published:** 2021-06-16

**Authors:** Huan Du, Zhicheng Jiao, Junjie Liu, Wei Huang, Liangfa Ge

**Affiliations:** 1 Guangdong Key Laboratory for Innovative Development and Utilization of Forest Plant Germplasm, College of Forestry and Landscape Architecture, South China Agricultural University, Guangzhou, 510642 Guangdong China; 2 Guangdong Engineering Research Center for Grassland Science, South China Agricultural University, Guangzhou, China; 3 State Key Laboratory for Conservation and Utilization of Subtropical Agro-Bioresources, College of Life Sciences, South China Agricultural University, Guangzhou, 510642 China; 4 The Guangdong Subcenter of the National Center for Soybean Improvement, College of Agriculture, South China Agricultural University, Guangzhou, 510642 China

**Keywords:** Fast neutron bombardment, *Medicago truncatula*, Next-generation sequencing, Mutation

## Abstract

**Background:**

Fast neutron bombardment (FNB) is a very effective approach for mutagenesis and has been widely used in generating mutant libraries in many plant species. The main type of mutations of FNB mutants are deletions of DNA fragments ranging from few base pairs to several hundred kilobases, thus usually leading to the null mutation of genes. Despite its efficiency in mutagenesis, identification of the mutation sites is still challenging in many species. The traditional strategy of positional cloning is very effective in identifying the mutation but time-consuming. With the availability of genome sequences, the array-based comparative genomic hybridization (CGH) method has been developed to detect the mutation sites by comparing the signal intensities of probes between wild-type and mutant plants. Though CGH method is effective in detecting copy number variations (CNVs), the resolution and coverage of CGH probes are not adequate to identify mutations other than CNVs.

**Results:**

We report a new strategy and pipeline to sensitively identify the mutation sites of FNB mutants by combining deep-coverage whole-genome sequencing (WGS), polymorphism calling, and customized filtering in *Medicago truncatula*. Initially, we performed a bulked sequencing for a FNB *white nodule* (*wn*) mutant and its wild-type like plants derived from a backcross population. Following polymorphism calling and filtering, validation by manual check and Sanger sequencing, we identified that *SymCRK* is the causative gene of *white nodule* mutant. We also sequenced an individual FNB mutant *yellow leaves 1* (*yl1*) and wild-type plant. We identified that *ETHYLENE-DEPENDENT GRAVITROPISM-DEFICIENT AND YELLOW-GREEN 1* (*EGY1*) is the candidate gene for *M. truncatula yl1* mutant.

**Conclusion:**

Our results demonstrated that the method reported here is rather robust in identifying the mutation sites for FNB mutants.

**Supplementary Information:**

The online version contains supplementary material available at 10.1186/s13007-021-00765-y.

## Introduction

Plant mutant collections are very useful genetic resources and widely used in forward and reverse genetic studies. Mutants can be induced by different mutagens, such as chemical agents or biological factors. Ethyl methane sulfonate (EMS) and Transfer DNA (T-DNA) are two popular mutagens that typically induce point nucleotide substitution and T-DNA insertional mutation, respectively [1–4]. Although EMS mutation is relatively easy to be generated, it always induces numerous background mutations and thus requires substantial efforts to identify causative mutation sites. By contrast, T-DNA insertion mutations typically harbor fewer background mutations and the mutation sites could be identified by the flanking sequences theoretically. However, T-DNA mutation requires the procedure of plant tissue culture and usually takes a long period to accumulate a large number of mutants’ collections.

FNB uses high-energy neutrons to irradiate plant seeds and mainly induces random deletions of various sizes on chromosomes. Because deletions likely cause either complete/partial loss of the corresponding genome fragments or induce frame-shift mutations, FNB is a very powerful mutagen to generate null mutations. In addition, the background mutations are much less in FNB mutants as compare to the EMS mutants, making it easier for further causative gene cloning work. Hence, FNB has been broadly used in creating mutant libraries in many model and crop plants, including *Arabidopsis thaliana*, *M. truncatula*, rice (*Oryza sativa*), and soybean (*Glycine max*) [5–7].

Despite the efficiency of FNB in mutating plant genomes, it is still challenging to identify the mutation sites. The classical positional-cloning method certainly could locate the candidate gene, it requires a segregating population derived from the intercross between the FNB mutant and another accession, and complex genetic linkage analysis for the molecular markers with mutation site [8, 9]. Consequently, it could take years to identify the candidate mutation [8, 9]. To accelerate the process of mutation identification, few new methods including CGH and Deletion-TILLING (De-TILLING) have been developed [6, 10]. In addition, Ge et at. (2016) reported a successful case using the Affymetrix microarray-based expression profiling dataset to identify the causative DNA deletion in *M. truncatula* [11]. These new methods were principally based on the DNA fragment deletion of substantial size that could lead to the decrease of DNA hybrid signal compared with wild-type or down-regulation of few adjacent genes, the limitation mainly lies in the relatively low resolution and accuracy. Along with the development of next-generation sequencing technology, several new algorithms/tools have been developed to detect DNA fragment deletions of various sizes, such as Pindel [12], BreakDancer [13, 14] and FNBtools [15]. FNBtools is particularly specialized for FNB mutants. It took advantage of the CIGAR information from the informative reads extracted from the short reads alignment files to locate the DNA breaking point and used a segregating population to detect the linkage between the deletion and the mutant phenotype.

These new methods continually improve the accuracy and reliability in predicting the structural variations on chromosomes. However, due to the complexity of genomes, particularly the duplications resulted from the whole genome duplication, we found that it is still challenging in detecting reliable mutations in many cases. In some cases, the reported deletions reflected false positives. In this work, we hypothesized that small deletions in FNB mutants can be viewed as polymorphic sites between mutants and wild-type plants, while large-size deletions can be viewed as presence-absence variations (PAV), which typically show extremely low or even no reads coverage in mutants, in contrast to the parallel datasets of controls which should display normal reads coverage. Based on this hypothesis, we developed a straightforward pipeline to detect the mutation sites of FNB mutants by combining the variant-calling pipeline and customized filtering of the variants. To establish the linkage between the identified mutation and the mutant phenotype, we applied the pipeline to the homozygous mutant and wild-type like plants derived from an F_2_ population and identified the mutated gene responsible for the phenotype.

## Result

### The pipeline for calling and filtering deletions of FNB mutants in M. truncatula

In order to identify the associated deletions linked to the mutants’ phenotypes, the mutants are usually backcrossed to the wild-type plants. Backcrossing not only purifies the background, but also creates a segregating population that can be used for Bulked Segregant Analysis coupled with Whole-Genome Sequencing (BSA-Seq) [16, 17]. For BSA-Seq, if the F_1_ plants show wild-type like phenotype and the ratio of mutant to wild-type like phenotype is 1:3 in F_2_ progeny, then the mutant is likely to be recessive. In this scenario, the mutant plants are pooled for DNA sequencing. The wild-type like plants, which are a mixture of both homozygous and heterozygous genotype, are also pooled for DNA sequencing. If F_1_ plants do not show wild-type phenotype and the ratio of mutant phenotype to wild-type like phenotype is significantly away from 1:3, the mutant is likely not to be recessive. In this scenario, F_3_ generation is used to identify homozygous mutant and wild-type like plants, which do not display phenotypic segregating in F_3_ progeny. The homozygous mutants are pooled for sequencing, as well as the wild-type like plants.

In case, if urgent identification of candidate deletions is needed and segregating population is not available, the individual FNB mutant plants are directly pooled and sequenced. In addition, the wild-type plants are also pooled and sequenced.

**Fig. 1:**
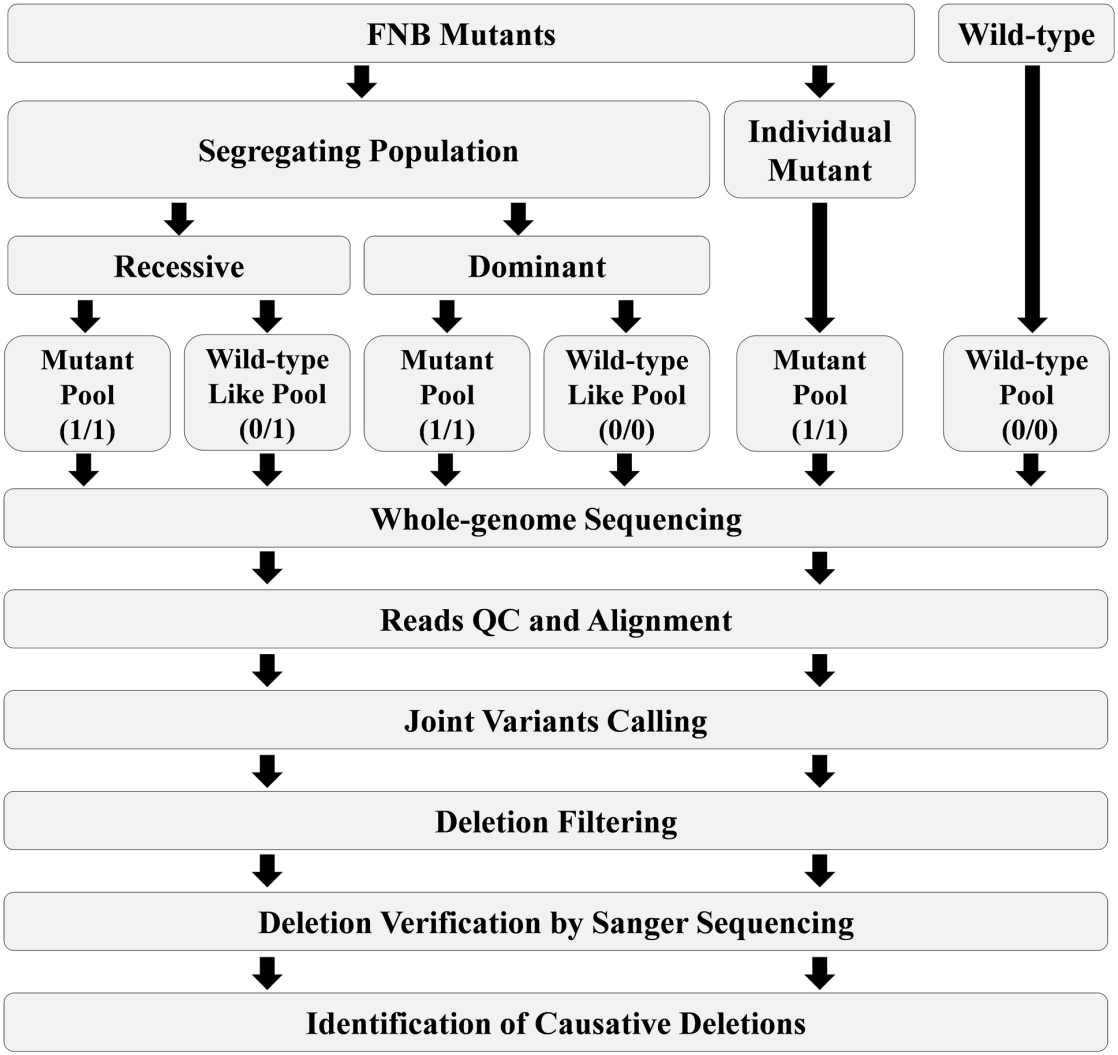
The step-by-step workflow diagram to identify candicate causative deletions for FNB mutants. FNB mutants, either from a segregating population or individual mutant, were sequenced by NGS. The wild type plants, and the wild-type like plants from the same segregating population of FNB mutants were sequenced as well. After the joint variants calling and fitering, deletions were verified by Sanger sequencing, and the causative deletion was identified. The numbers in the brackets represet the genotypes (0/0: homozygous wild type; 0/1: heterozygous; 1/1: homozygous mutant)

**Fig. 2:**
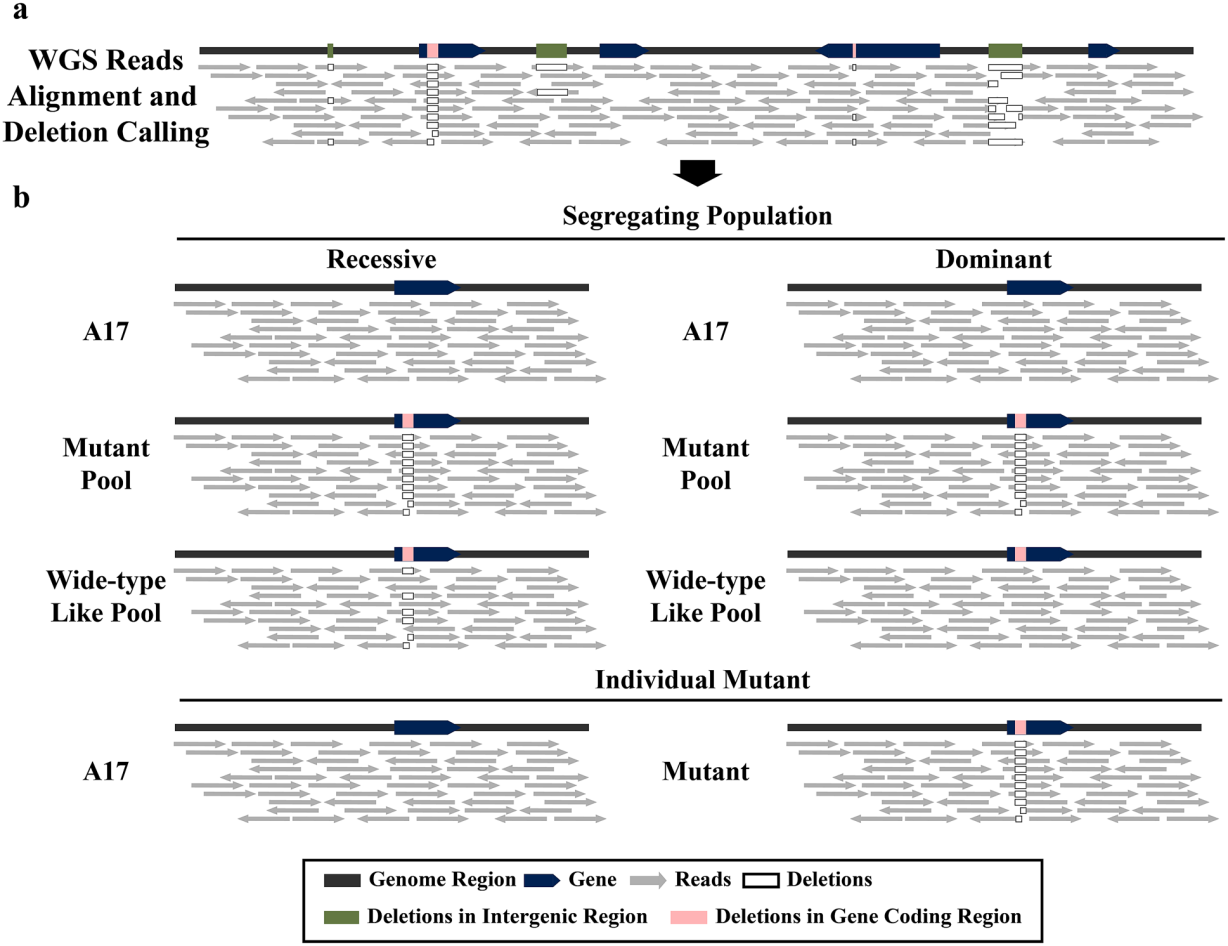
The pipeline of calling and filtering deletions. **a** Illustration of the reads alignment and deletion calling steps for FNB mutant NGS data. **b** Identification of causative deletions for FNB mutants from segregating populations. **c** Identification of candidate deletions for individual FNB mutant

To achieve adequate coverage, we propose at least a 20 × depth of the Illumina paired-end sequencing. After the quality control procedures for the raw reads, a variant discovery calling pipeline is used to identify the mutations of the FNB mutants (Figs. 1 and 2). We recommend the GATK pipeline for a parallel calling for a cohort of independent FNB mutants simultaneously, which would contribute to filter the background mutations [18–21]. If the mutant is recessive, the genotype of the mutant pool is a homozygous mutation, whereas the genotype of the corresponding wild-type like plants pool is heterozygous mutation. In contrast, wild-type plants and other irrelevant mutants are all wild-type genotype. If the mutant is not recessive, the mutant pool is a homozygous mutation, and wild-type pool, which is made of the wild-type like plants that do not show phenotypic segregating in F_3_ generation, is a homozygous wild-type genotype. The wild-type plants and other irrelevant mutants are also homozygous wild-type genotype. For the individual mutant, the mutant pool is a homozygous mutation, whereas all other pools and wild-type plants are wild-type genotypes. Based on these criteria, the variants are filtered for each mutant. The deletions that pass the filtering are further compared with the genome annotation file. If the mutations fall within the gene coding regions, they can be considered candidate mutations. Additionally, the short reads alignment files, such as BAM files, are used to visually confirm the mutations. Finally, the candidate deletion borders are amplified and further confirmed by Sanger sequencing.

### A case study, identification of the causative gene for an FNB mutant using FNB-BSA-Seq in M. truncatula

**Fig. 3:**
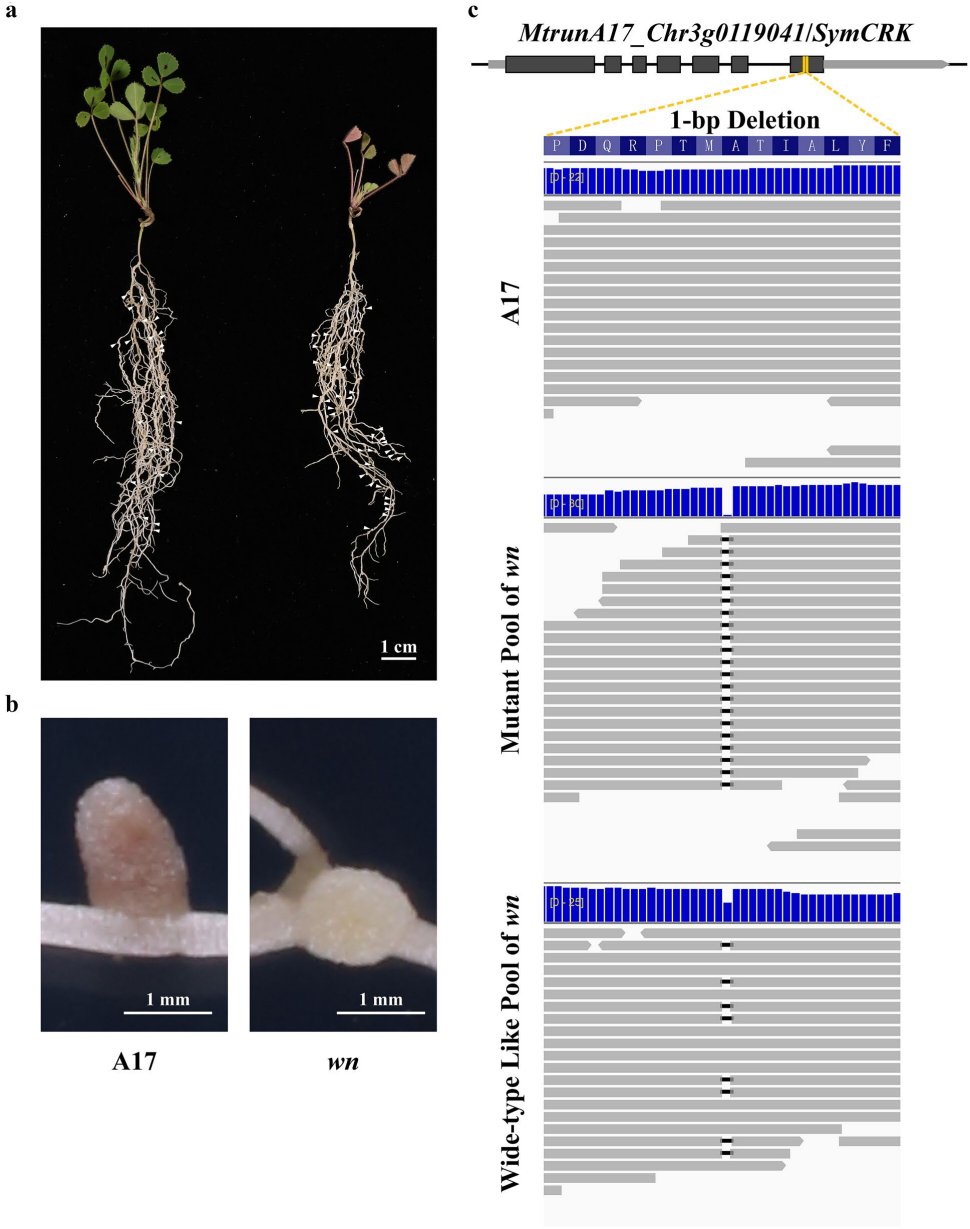
The phenotype and reads alignment of *wn* mutant. **a** The phenotype of whole plants for A17 (left) and *wn* mutant (right). **b** The nodule phenotype for A17 (left) and *wn* mutant (right). **c** The reads alignment for A17, mutant pool of *wn* and wide-type like pool of *wn*

To evaluate the effectiveness of the pipeline and identify the causative gene for a fixation-minus (fix^–^) FNB mutant, which developed white and small nodules, named as *white nodule* (*wn*) (Fig. 3a, b), we applied BSA-Seq pipeline to the *wn* mutant. *wn* was first backcrossed to wild-type *M. truncatula* (cv. Jemalong A17). In the F_2_ generation, 14 mutant plants that developed fix^–^ nodules and 46 wild-type like plants that produced normal nodules were observed. The chi-square test indicated that the mutant to wild-type segregation ratio fits 1:3, suggesting that *wn* is a single recessive mutation. The mutant plants were all pooled for WGS. As the control, 25 wild-type like plants were also pooled for WGS, as well as the wild-type plant A17 that can serve as an additional control. For the mutant pool, more than 42.6 million paired-end reads (2 × 150 bp) were produced, which represents a 29.7 × sequencing depth (Table 1). For the wild-type like plants pool and A17 pool, 48.8 and 43.1 million reads were produced, representing 34.1 × and 30.1 × sequencing depth respectively (Table 1). The reads were mapped to the latest reference genome of *M. truncatula* (MtrunA17r5.0) [22] and the cohort polymorphisms were called using the mapped reads. Since the mutant is recessive, the causative mutation must be homozygous deletion, whereas the pool made of the wild-type like plants should be heterozygous genotype, and A17 plants should be wild-type genotype. On the basis of this guideline, the identified deletions were further filtered. In the mutant pool, totally nineteen homozygous deletions, spanning one to eighteen base pairs, were found, and the corresponding genotypes of the wild-type like plants pool and A17 were heterozygous and wild type, respectively.

**Table 1:** Statistics of short reads and sequencing depth

	A17	*wn* Mutant Pool	*wn* Wild-type Like Pool	*yl1*
Reads number	43,067,006	42,571,812	48,823,767	57,358,177
Sequencing depth	30.1x	29.7x	34.1x	40.1x

**Fig. 4:**
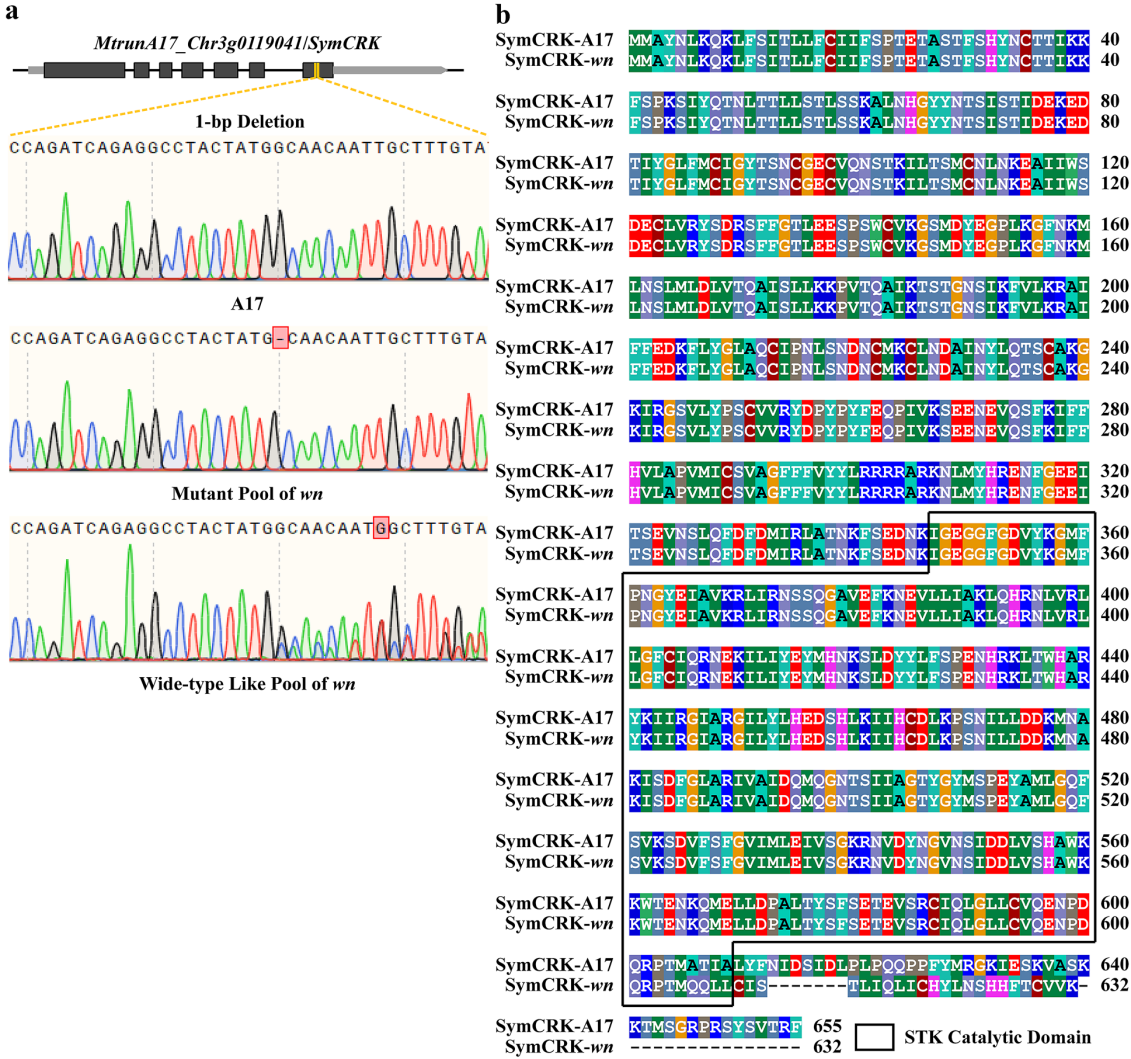
The alignment of DNA and protein sequence of A17 and *wn* mutant. **a** The Sanger sequencing result of A17, mutant pool and wide-type like pool, respectively. **b** The SymCRK protein sequence alignment of A17 and *wn* mutant. Black block indicated the conserved STK catalytic domain of SymCRK protein

The deletions were further visually confirmed by visualizing the short reads alignment details in Integrative Genomics Viewer (IGV) [23]. Among the nineteen deletions, sixteen are located in the intergenic regions, and two are located in the untranslated regions (UTRs) and do not affect the coding sequences. The only one located in the coding region is a single base pair deletion, which caused the frameshift of *MtrunA17_Chr3g0119041* (Fig. 3c and Additional file 2: Table S1). The deletion was further confirmed by Sanger sequencing (Fig. 4a). *MtrunA17_Chr3g0119041* encodes a Serine/Threonine Kinase (STK), also termed as SymCRK in a previous report [24] (Fig. 4b). It has been documented that *SymCRK* controls the senescence process of nodules in *M. truncatula*. The deletion in the coding region of *SymCRK* caused the defect in the STK catalytic domain. Similar to *wn* mutant, *symcrk* mutant also produced white and necrotic nodules [24]. These results suggested that 1-bp deletion in *SymCRK* caused the defective nitrogen fixation phenotype of *wn* mutant, reflecting that *SymCRK* was the causative gene of *wn* mutant.

### A case study, identification of the causative deletion for an individual FNB mutant in M. truncatula

**Fig. 5:**
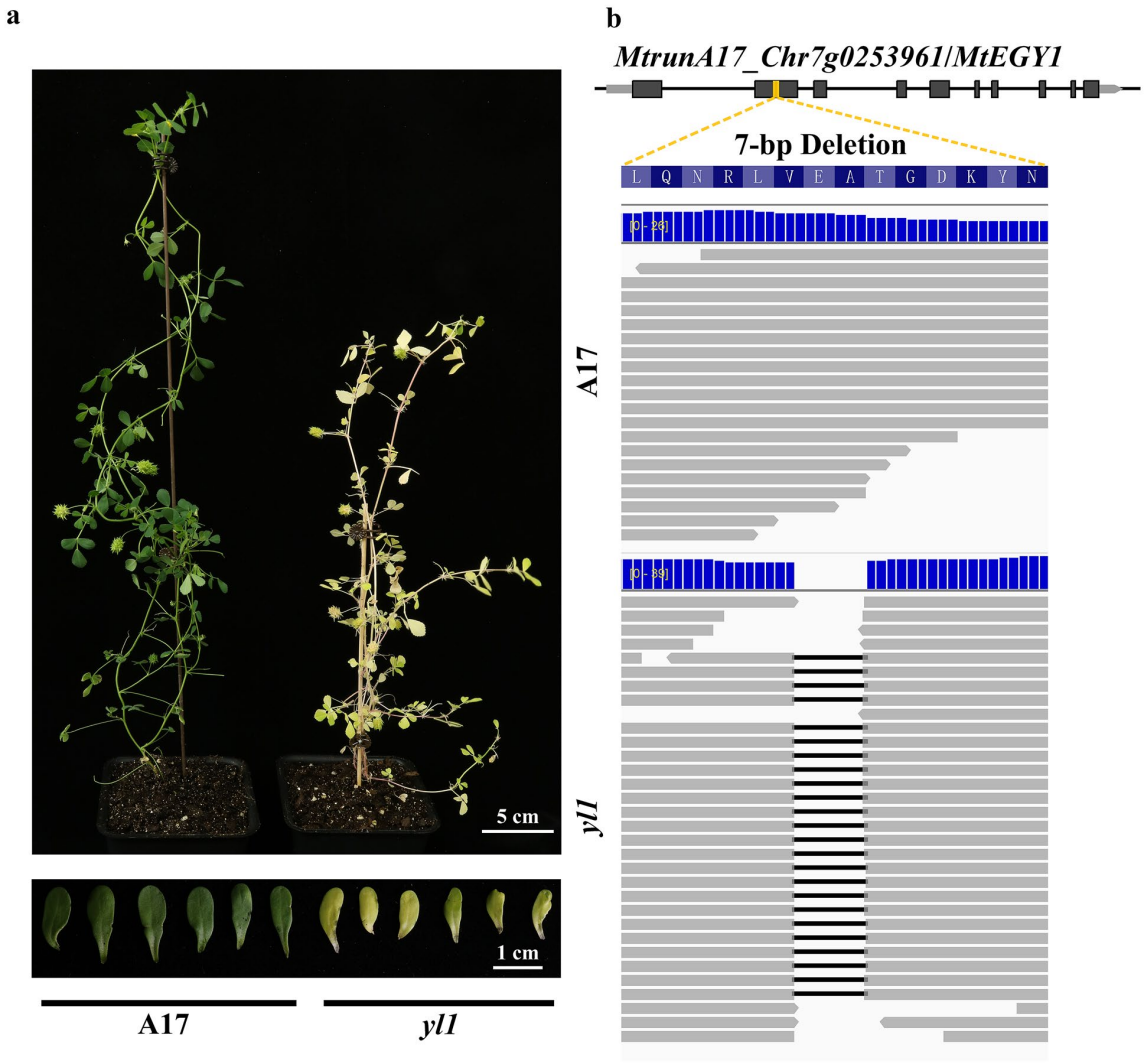
The phenotype and reads alignment of *yl1* mutant. **a** The phenotype of whole plants and cotyledon for A17 (left) and *yl1* mutant (right). **b** The reads alignment for A17 and *yl1* mutant

To evaluate the effectiveness of the pipeline for individual FNB mutants, we applied the pipeline to an FNB individual mutant, which developed yellow leaves and was named *yellow leaves 1* (*yl1*)*.* Phenotypic analysis indicated that the chlorophyll concentration of *yl1* mutant was significantly lower than A17 plants, resulting in yellow-green cotyledon and leaves (Fig. 5a).

To rapidly identify the possible causative gene for *yl1*, *yl1* plants were pooled and directly sequenced by WGS. Totally 57.4 million paired-end reads were produced for *yl1*, representing a 40.1 × sequencing depth (Table 1). For polymorphisms calling, both *yl1* and A17 pools were called simultaneously. The raw polymorphism data were first filtered by the quality, followed by the genotype. In the filtering process, we detected a large deletion which is about one kilobase pair (kb). This large deletion presents in both *yl1* and A17, thus it was not considered a candidate deletion (Additional file 1: Fig. S1). The deletions that were only presented in *yl1* pool were considered potential candidates. In total, 115 deletions existed in *yl1* pool only. Among these deletions, two were located in the coding region of genes on chromosome 7, and caused the frameshift mutation for the corresponding genes (Additional file 3: Table S2).

**Fig. 6:**
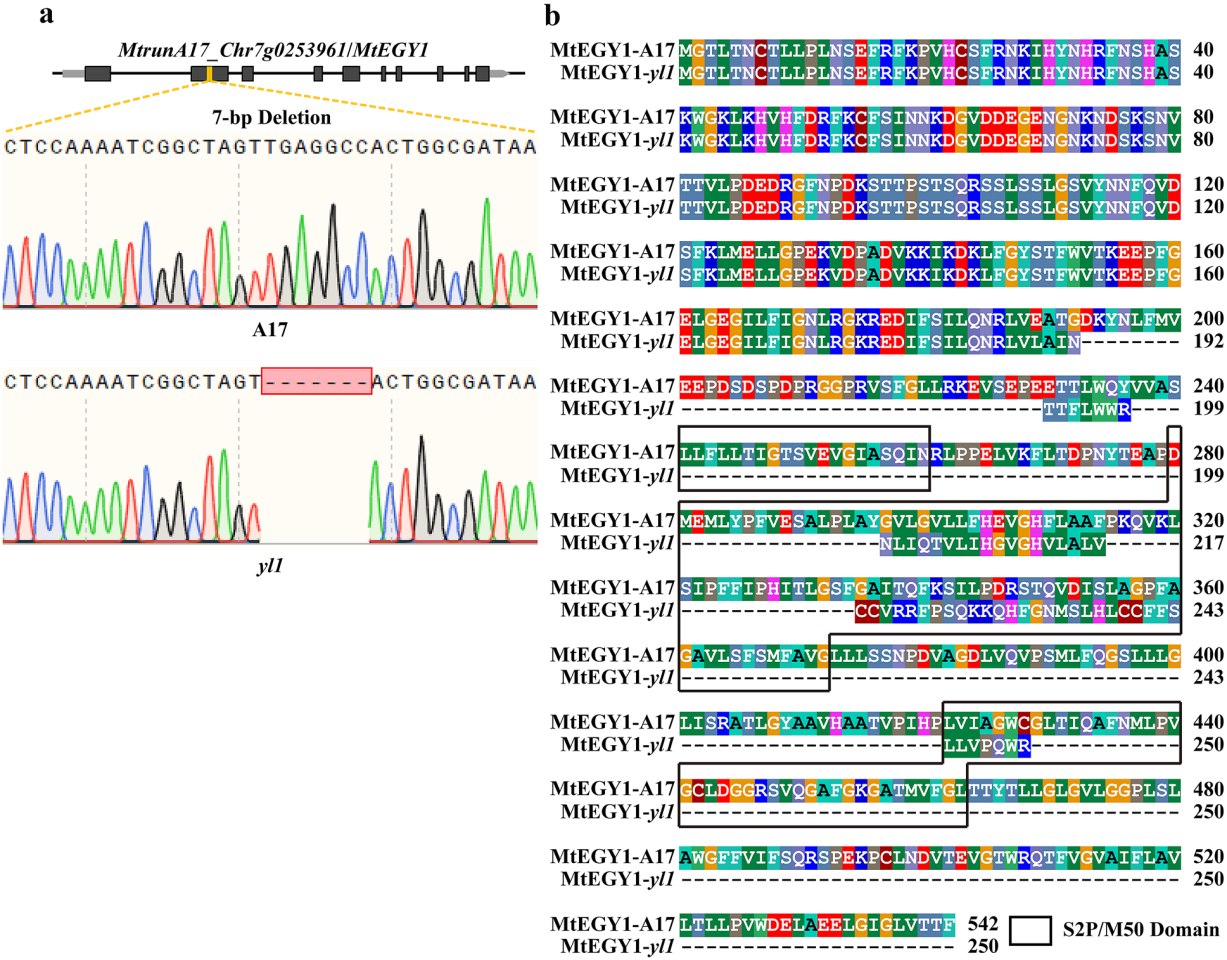
The alignment of DNA and protein sequence of A17 and *yl1* mutant. **a** The Sanger sequencing result of A17 and *yl1* mutant. **b** The alignment of MtEGY1 from A17 and *yl1* mutant. Black block indicated the conserved S2P/M50 domain of MtEGY1 protein

Visualization of the short reads alignment files in IGV indicated that the candidate deletions were well supported by the reads alignment (Fig. 5b). One of the candidate deletions is located in *MtrunA17_Chr7g0223481*, which encodes a putative stigma-specific protein. The 1-bp deletion caused the frameshift of *MtrunA17_Chr7g0223481*. *MtrunA17_Chr7g0223481* is the homolog of *At1g50650* in *Arabidopsis thaliana*, which encodes the STIG1 family peptide KERBEROS (KRS). KRS regulates the development of embryo sheath [25], and *krs* mutant showed the absence of sheath production by endosperm [25]. Another deletion was located in the second exon of *MtrunA17_Chr7g0253961*, which encodes a putative peptidase M50 protein (Fig. 5b). The 7-bp deletion caused frameshift mutation in *MtrunA17_Chr7g0253961* and the loss of S2P/M50 (Site-2 protease/zinc metalloproteases) domain, which is critical for the proper function of the protein (Fig. 6a, b). Based on the homolog analysis, we found that the *MtrunA17_Chr7g0253961* is the homolog of *At5g35220* in *A. thaliana*, which encodes ETHYLENE-DEPENDENT GRAVITROPISM-DEFICIENT AND YELLOW-GREEN (EGY1). A previous report documented that EGY1 is required for the development of chloroplast and accumulation of chlorophyll in Arabidopsis [26]. The *A. thaliana egy1-1* mutant developed yellow-green leaves, which similar to the leaves of *yl1* mutant. Thus, it is highly likely that the *EGY1* homologous gene, *MtrunA17_Chr7g0253961*, is the causative gene of *yl1*.

## Discussion

Mutagenesis is a very important tool to dissect genes’ function in plant genetic research. FNB, which employs high-energy neutron as the mutagen, is very effective in generating various deletions on the chromosomes. Owing to its mutagenesis efficiency in generating null mutations, FNB has long been used to create large-scale genetic resources for forward and reverse genetics studies, and even used as a breeding technique for many species. Due to the complexity of plant genomes, identifying the causative deletions for FNB mutants has always been challenging in many studies, particularly before the era of NGS. Positional cloning was one of the most popular methods to identify the causative gene for FNB mutants of interest. However, it was rather time-consuming. In order to increase the efficiency in identifying the mutations, new approaches such as CGH and Deletion-TILLING have been developed [6, 27]. These new approaches provided new solutions in dealing with the FNB mutants. However, the effectiveness and accuracy are still to be improved for such probe or PCR-based methods [15]. These disadvantages have limited the utilization of FNB mutants as an invaluable genetic resource. The rapid progressing of NGS technology has greatly renovated the strategy in detecting the whole genome polymorphisms and offers many advantages over the traditional methods in identifying the mutations for FNB mutants. With continuous cost reduction, NGS has been a regular tool and accessible to most researchers. WGS by NGS for FNB mutants not only increases the sensitivity in detecting mutations on chromosomes, but also improves the reliability and resolution in identifying deletions. In this work, we developed a useful and straightforward pipeline for the identification of candidate deletion for FNB mutants in *M. truncatula*. The pipeline takes full advantage of high-depth WGS and advanced deletion-calling algorithm and presents a viable solution in identifying the candidate causative genes for the FNB mutants.

The pipeline developed by this work is effective in identifying the causative deletions for FNB mutants, as demonstrated by the application of pipeline to the two representative FNB mutants, one in a segregating population and another in individual FNB mutant. FNB-BSA-Seq has detected a single base pair deletion in *SymCRK* coding sequence of *wn* mutant. The 1-bp deletion in *wn* mutant caused frameshift null mutation of *SymCRK* and led to white and necrotic nodules, which resembles the phenotype of *symcrk* mutant. The 1-bp deletion is rather small and would not be identified by the probe-based approaches. Combining 30 × deep sequencing and deletion calling/filtering pipeline, the 1-bp deletion of *wn* mutant stood out with high-quality scores in the report of the pipeline, demonstrating that the pipeline is very effective in identifying small deletions of FNB mutant.

For *yl1* mutant, there is no segregating population, thus we are unable to establish the linkage between the deletion and mutant. However, a mutant without a useful segregating population is very common in the practices of genetic researches, as generating a segregating involves multiple steps and progenies, and usually takes a long time, especially for those plants with long life cycles (*M. truncatula* typically has a life cycle from four to six months). It would be very useful if the candidate deletions could be identified for the individual FNB mutant. In our practice, the pipeline reported only two candidate deletions that are located in the coding region of genes. This is probably because that FNB mutant usually has a less background mutation compared with the mutants induced by other chemical mutagens like ethyl methanesulfonate (EMS) [6]. The two candidate deletions of *yl1* mutant are 1-bp and 7-bp respectively, both causing the frameshift in the coding sequences. Through protein homologs and annotation searches, we found that the deletion in *MtrunA17_Chr7g0223481* is likely the causative mutation of *yl1*, since *MtrunA17_Chr7g0223481* is the homolog of Arabidopsis *EGY1*, whose mutation led to the similar phenotype of *yl1*. Taken together, the example of *yl1* mutant demonstrated not only the effectiveness of the pipeline in identifying the deletion for the FNB mutants, but also the possibility of application of the pipeline to the individual FNB mutant for causative gene cloning.

WGS uses high coverage short reads to examine the homogeneity between the reference genome and short reads. Sequencing depth is an important parameter that determines the sensitivity and reproducibility in detecting variations among genomes [28, 29]. In this work, we proposed a minimum of 20 × sequencing depth for mutation detection. Given sufficient sequencing depth, WGS could theoretically detect all potential deletions caused either by artificial mutagenesis or natural variation, which has driven the utilization of WGS for indel calling in many studies. WGS usually generates a large-scale dataset including tens of millions of raw short reads, which require well-designed algorithms and intense data analyzing procedures to extract the polymorphic information. During the past decade, there are a few algorithms developed to detect the deletions from the WGS data [12–15]. These algorithms/tools investigate the deletions on chromosomes from different angles, including using informative reads to calculate the deletions or reads coverage survey. In this work, we treated the small deletions of FNB mutant as the indel sites, which widely exist in the natural populations and have been tensely studied in recent years. There have been successful tools developed to identify such small indels, including GATK4’s HaplotypeCaller. Although it is not designed for FNB mutants, HaplotypeCaller performs well in terms of sensitivity and accuracy, possibly owing to its unique ability of de-novo local assembly of haplotypes for the regions where a potential polymorphism occurs [21]. GATK4’s variant discovery workflow is well documented and maintained by Broad Institute. Applying this workflow to the WGS dataset of FNB mutants is straightforward and requires a minimal learning process, making it feasible for most researchers to rapidly identify the candidate deletions for FNB mutants.

## Conclusion

Despite the progress in developing methods to identify deletions for FNB mutants, it remains challenging to rapidly isolate the causative mutations in many studies. Combining WGS, the variant discovery workflow, and filtering by comparing the genotype of FNB mutants and control lines, we developed a simple but efficient pipeline to rapidly identify the candidate causative deletions for FNB mutants in *M. truncatula*. As demonstrated by the two case studies, the pipeline combines sensitivity and accuracy in detecting mutations, and the filtering process by comparing with multiple controls is very useful to pinpoint the causative mutations. FNB mutant collections are available for many model and crop species. Due to the difficulty in rapidly identifying the causative genes, these invaluable resources have not been fully utilized. The principle and pipeline described here can also be applied to the FNB mutants of other species, offering a reliable solution to utilize FNB mutants for genetic research or crop breeding.

## Methods

### Plant materials

The *M. truncatula* plants were germinated in a petri dish and placed at 4 ℃ for one week. After germination, the plants were grown in a growth chamber at 22 ℃/16 h light and 20 ℃/8 h dark. To generate the F_2_ segregating population, *wn* mutant was backcrossed to *M. truncatula* cv. Jemalong A17 and F_1_ plants were selfed to generate the F_2_ population. The cotyledon and leaf phenotypes of *yl1* and A17 were analyzed one month and two months post-germination respectively.

### Rhizobia inoculation and nodule phenotype analysis

The F_2_ population of *wn* mutant was grown in sand and inoculation with rhizobia *Sm2011* (*Sinorhizobium meliloti 2011*) as previously reported [30]. The nodules of wide-type and *wn* were observed and analyzed with the stereomicroscope after 30 days post-inoculation. The plants with white and necrotic nodules were pooled as the mutant pool, and the plants with red and normal nodules were pooled as the wild-type like pool.

### DNA extraction and sequencing

The DNA samples from leaves were extracted using the Trelief ™ Plant Genomic DNA Kit (Beijing TsingKe Biotech; TSP101). The DNA samples were sequenced on an Illumina Nova-seq platform with a 150-bp paired-end (PE) protocol.

### Sequence alignment, indel calling, and visualization

The polymorphism calling was conducted according to GATK4’s (GATK v4.1.7.0) instruction [19]. Briefly, the 150-bp pair-end reads were mapped to the *M. truncatula* reference genome (MtrunA17r5.0) using the BWA-MEN (Burrows–Wheeler Aligner; Version 0.7.12-r1039) [31]. For reads alignment, mem algorithm was used with a minimum seed length of 19, band width of 100, and off-diagonal X-dropoff of 100. After sorting, adding reads groups, validating, and marking for duplicates, the mapped reads in bam format were used to call variants by HaplotypeCaller in GVCF mode [20]. For the calling step, the assembly-region-padding was set to 100, the base-quality-score-threshold was set to 19, the max-reads-per-alignment-start was set to 50, and the max-assembly-region-size and min-assembly-region-size were set to 300 and 50, respectively. The individual GVCF files were subjected to joint variant calling. The indels were filtered from the variants after calling using SelectVariants function of GATK. The deletions were selected from the indels for further analysis. The IGV (v2.8.2) software was used for further visual verification of deletions of the mapped reads [23].

### Sanger sequencing validation

The deletion borders of *SymCRK* in *wn* and *MtEGY1* in *yl1* were amplified using Q5® High-Fidelity DNA Polymerase (NEB #M0491) with primers spanning the target deletion sites. The PCR was conducted as follows: 98 °C for 10 s, 60 °C for 30 s and 72 °C for 30 s for 35 cycles. The PCR products were Sanger sequenced. The primers were listed in the Additional file 4: Table S3.

## Supplementary Information

**Additional file 1: Figure S1** The identified large fragment deletion of *yl1* mutant. (a) The coordinate and size of the large deletion identified in both A17 and *yl1* mutant. (b) Visualization of aligned reads surroungding the deletion in both A17 and *yl1* mutant.

**Additional file 2: Table S1.** The potential deletions in *wn* mutant. The details of potential deletions in *wn* mutant populations were listed, which include the location, sequence, length, genotype in different samples and the mutant type of deletions.

**Additional file 3: Table S2.** The potential deletions in *yl1* mutant. The details of potential deletions in *yl1* mutant populations were listed, which include the location, sequence, length, genotype in different samples and the mutant type of deletions.

**Additional file 4: Table S3.** The list of primers. The details of primers for genotyping of *SymCRK* and *MtEGY1* were listed.

## Data Availability

The raw NGS data generated in this study are deposited into NCBI database under the accession number PRJNA714779.

## Abbreviations

FNB: Fast neutron bombardment
CGH: Comparative genomic hybridization;
CNVs: Copy number variations
WGS: Whole-genome sequence
EMS: Ethyl methane sulfonate
T-DNA: Transfer DNA
De-TILLING: Deletion-TILLING
BSA-seq: Bulked segregant analysis coupled with whole-genome sequencing
IGV: Integrative genomics viewer
NGS: Next-generation sequencing
