# Supplementary figures and images for: Rapid identification of mutations caused by fast neutron bombardment in Medicago truncatula
Source: Plant Methods. 2021 Jun 16;17:62. doi: 10.1186/s13007-021-00765-y (PMC8207604; doi:10.1186/s13007-021-00765-y)

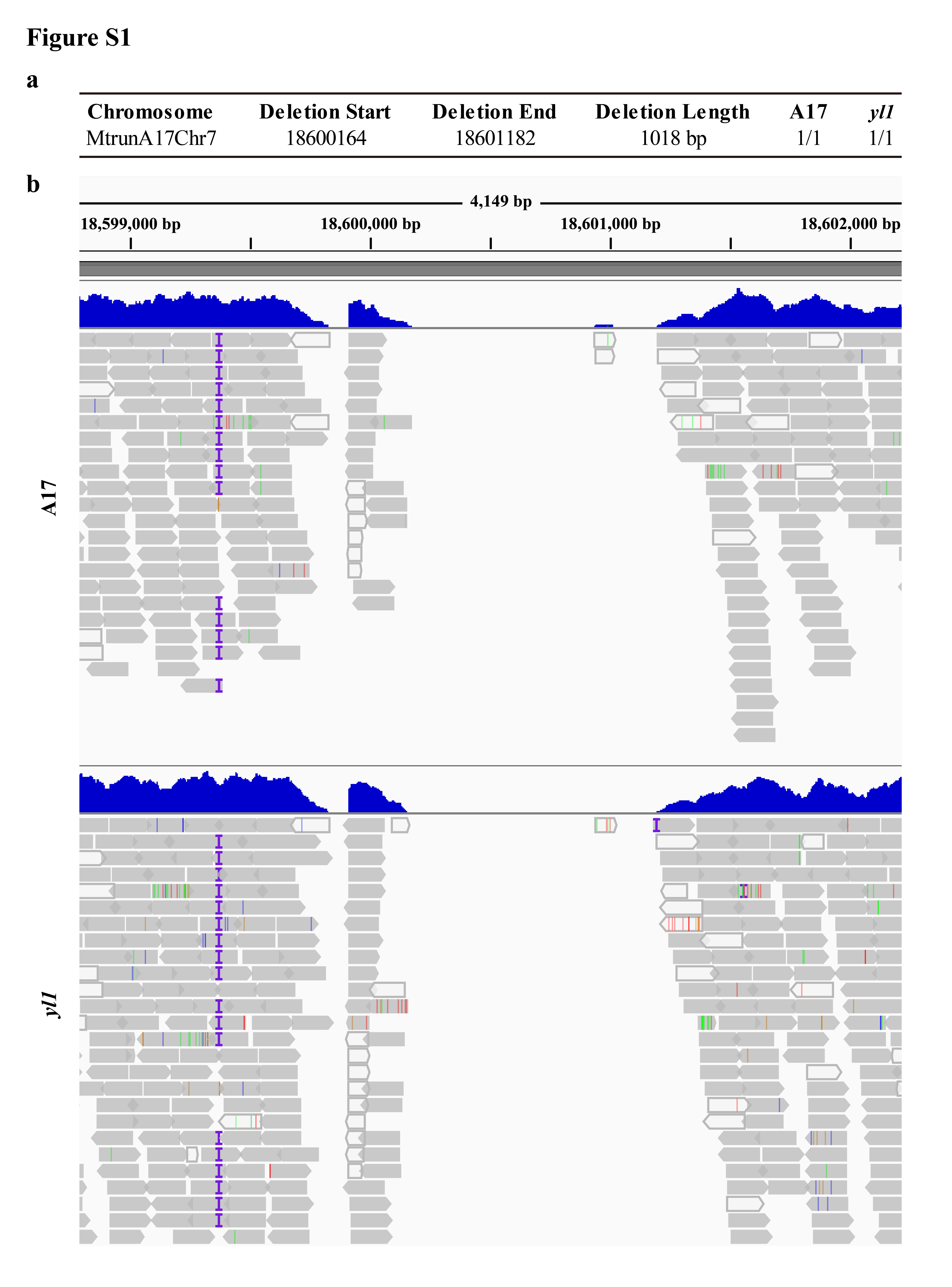

Supplement: Supplementary file 1 — Additional file 1: Figure S1 The identified large fragment deletion of yl1 mutant. (a) The coordinate and size of the large deletion identified in both A17 and yl1 mutant. (b) Visualization of aligned reads surroungding the deletion in both A17 and yl1 mutant. [file 13007_2021_765_MOESM1_ESM.tif]
